# Evaluation of the Criteria for Quality of Life of Elderly Health Care Centers in Tehran Province, Iran

**DOI:** 10.5539/gjhs.v8n7p68

**Published:** 2015-11-03

**Authors:** Fereshteh Farzianpour, Mohammad Arab, Abbas Rahimi Foroushani, Esmaeil Morad Zali Mehran

**Affiliations:** 1 Department of Health Management and Economics, School of Public Health, Tehran University of Medical Sciences, Tehran, Iran; 2 Departments of Epidemiology and Biostatistics, Tehran University of Medical Sciences, Tehran, Iran

**Keywords:** Quality of Life, elderly, SF-36 questionnaire, healthcare centers, Tehran

## Abstract

**Background and Objectives:**

The objective of this study was to evaluate the elderly quality of life of people covered by the healthcare centers in Tehran and its influencing demographic and background factors.

**Method:**

This is a cross-sectional study of quality of life of the elderly population covered by healthcare centers and bases in Tehran, as well as the influential background and demographic factors. Sampling was performed using simple random stratified sampling proportionate to the size of strata. Data were collected using the Iranian version of the standard questionnaire Short Form Health Survey (SF-36).

**Results:**

According to the findings, 240 (60%) of the cases were men and 160 (40%) were women. Regarding age distribution, 76.3% fell in the 60-69 age range and 87.2% were illiterate. 18% of the elderly stated that they have financial problems and 19.5% did not express any financial problems. While studying the relationship between financial status and health status with the mean scores of quality of life, statistically significant differences were observed in all domains (p=0.032<0.001). The mean quality of life was lower in women compared to men.

**Conclusions:**

The findings of the present study indicate that the health-related quality of life in the elderly population is influenced by their health status and demographic and background variables.

## 1. Background

The successful implementation of birth control programs, enhancement of primary healthcare services, improvement of socioeconomic status and development of novel technologies for prevention, diagnosis and treatment of diseases have contributed to an increase in the number of people aged 60 years of more, defined by the World Health Organization as the elderly population (Farzianpour et al., 2012a). Currently, there are more than 600 million elderly individuals in the world; a figure which will double by 2025 and will reach 2 billion people by 2050 (Malekafzali et al., 2010; Farzianpour et al., 2015a). By 2030, the number of U.S. adults aged 65 years or older wills more than double to about 71 million. The rapidly increasing number and diversity of older Americans has far-reaching implications for the U.S. public health system and will place unprecedented demands on aging services and the nation’s entire health care system. For example, Medicare spending has grown about nine-fold in the past 25 years, increasing from $37 billion in 1980 to $336 billion in 2005. If left unchecked, health care spending will increase 25% by 2030, largely because of the aging population. Chronic diseases disproportionately affect older adults and are associated with disability, diminished quality of life, and increased costs for health care and long-term care. Today, about 80% of older adults have at least one chronic condition, and 50% have at least two. These conditions can cause years of pain and loss of function. Public health efforts can help Americans avoid preventable illness and disability as they age. Research has shown that poor health is not an inevitable consequence of aging. Effective public health strategies currently exist to help older adults remain independent longer, improve their quality of life, and potentially delay the need for long-term care (http://www.cdc.gov/aging). Foreign researchers have reported, for example a reasercher from America showed Association between older age and more successful aging: Critical role of resilience and depression (Jeste and et al., 2013). Christina et al from Greece, The Healthy lifestyle and personal control questionnaire (HLPCQ): a novel tool for assessing self-empowerment through a constellation of daily activities (Christina et al., 2014). Bacsu et al. from Canada, healthy aging in place: Supporting Rural Seniors’ Health Needs (Bacsu et al., 2014). Woodard et al reported aging well and the environment: Toward an integrative Model and research agenda for the future (Woodard et al., 2012). The immense changes in cultural, socioeconomic and demographic aspects of Iranian society during the last few decades have given rise to an increase in the elderly population (Lutz et al., 2008). A review on the age structure of Iranian population during the last half century reveals the increase in number and percentage of elderly individuals (Lutz et al., 2008): The senescent population of Iran has almost doubled from 3.9% in 1956 to 7.3% (5121043 individuals) in 2006 (Lutz et al., 2008). The rapid, unprecedented growth of elderly population in developing countries, which are still in fight with poverty and contagious diseases, is posing an obstacle for development, thus necessitating urgent measures and approaches for remedy (Tajvar et al., 2004). In many of these countries, poverty, deficits in social security programs, continuous urbanization and ever-growing recruitment of women as workforce all compromise the traditional forms of caring for the elderly people (Tajvar et al., 2004). It is said that an aging population is the consequence of development; however, if we are unprepared for this phenomenon in a developed world, it will lead to many complications (Physical Activity Guidelines Advisory Committee Report, 2008). Therefore, the international community is paying greater attention to encountering the adverse outcomes of this phenomenon through adaption of appropriate policies in order to enhance the physical, mental and social status of the elderly population – i.e. improve their quality of life (White et al., 2009). While the major challenge of the twentieth century was “to survive”, the current century calls for “life with improved quality” (White et al., 2009). In Iran, however, only one percent of official regulations pertain to the elderly population, dealing with economic (62%), social (22%) and healthcare and welfare issues (16%) (Tajvar et al., 2004). This indicates that the elderly quality of life has not received due attention from Iranian authorities (Eshaghi et al., 2006). Therefore, we undertook this study to obtain some information regarding the elderly quality of life (Farzianpour et al., 2012b). The objective of this study is to determine the quality of life of the elderly population covered by healthcare centers in Tehran, so that this information may be used by healthcare authorities and executives to take up decisive steps to meet the needs of elderly individuals regarding their quality of life.

## 2. Methods

This research was approved by the Vice Chancellor for Research of Tehran University of Medical Sciences and the Research and Ethics Committee as #19430-123225on March 27, 2012. The study was an analytical cross sectional study with descriptive and analytical parts.

The population was individuals above 60 years of age (defined as elderly) in the province of Tehran, Iran. In the first phase, the number of elderly in healthcare centers in Tehran province was determined, N=23.345. Next, samples were selected from the population of the22 healthcare centers. For sampling, each healthcare center was considered to be a cluster for which simple random sampling was carried out and which were surveyed. The goal was to evaluate the elderly quality of life of people covered by the healthcare centers in the province of Tehran and its influencing demographic and background factors. The sample size was calculated based on a correlation coefficient; a correlation of .2 or greater is statistically significant at 80% for power of test at a 95% confidence level. As follows:


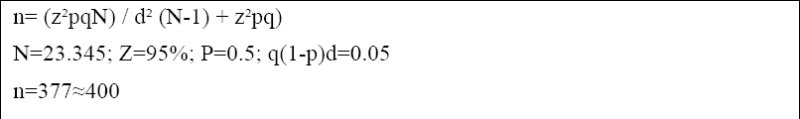


The sample size was multiplied by the ratio of cluster sampling (1.5), and Sample size was computed as 377. However, 400 (about (1.6%) elderly (aged 60-80) who met the eligibility criteria were selected from all 22 health centers.

### 2.1 Data Collection Methods and Tools

Data were collected using the SF-36 questionnaire to determine quality indicators. The results were analyzed using SPSS. V.17. The questionnaires consisted of two parts. The first part obtained demographic and other background information that is assumed to affect QoL and quality indicators of the elderly. The second part contained questions related to QoL. The SF-36 questionnaire scored all questions and the general QoL quantitatively on a scale from 0 to 100. The questionnaires were completed by interview and observation. The SF-36 is a general questionnaire which has been translated and used in over 50 countries, including Iran. The questionnaire involves two major scales: the physical component summary and the mental component summary, consisting of 8 subscales (domains) totally: physical functioning (PF), role physical (RP), bodily pain (BP), general health (GH), vitality (V), social functioning (SF), and role emotional (RE) and mental health (MH). Each dimension was scored from 0 through 100. The SF-36 has been validated by Montazeri et al (2005) as having a Cronbach’salpha coefficient of 0.95 for the Iranian population (Mohamadian et al., 2011; Montazeri et al., 2005). And reliability of the questionnaire was estimated using Cronbach’s alpha with a coefficient of 0.83 (Farzianpour et al., 2015b; Farzianpour & Tajvar, 2004). Using non-parametric Kruskal-Wallis test and multivariable regression.

### 2.2 Location of Research

This study was conducted on 400 individuals attending in the healthcare centers in Tehran province.

### 2.3 Ethical Considerations

All participants were given a full explanation of the study and freely consented to participate in the research. The questionnaires did not contain the names of the participants, and they were assured that the information collected would be kept confidential and under no circumstances would the published results contain the names of the participants.

### 2.4 Overcoming Operational Limitations

The limitations of this study included several changes in management of the State Welfare Organization of the province that delayed the implementation phase of the project. Other restrictions were the lack of cooperation by some elderly for completing the questionnaires, and it was necessary to fully explain all options to them. In some cases, educated and interested members of their households were asked to encourage and explain the importance of the project.

## 3. Results

Our study population consisted of 400 elderly individuals, ranging from 60-90 years of age, with a mean of 66.7 years and standard deviation of 8.2. Among these, 60% were men and 40% were women. Regarding education, 87.2% were illiterate, 10.2% had reached high school and 2.5% had achieved high school diploma. 3.8% of them lived alone and 72.7% lived with their partner and/or their children. 74.8% were married and 25.2% were unmarried (divorced or widowed). 18% stated that they were financially challenged and 19.5% mentioned no financial problems (Table 1). Our findings regarding the mean values of the couplet indices of quality of life, as measured from the questionnaire’s 0-100 scores, indicate a mean value of 53.2 for physical functioning, 31.6 for role physical, 51 for bodily pain, 47.1 for general health, 52.4 for vitality, 60 for social functioning, 40.9 for role emotional, and 61.4 for mental health. Regarding the overall scores of dimensions of quality life, the physical and mental components scored 45.7 and 53.7, respectively, yielding an overall score of 49.7 for the couplet indices of quality of life (Table 2).

**Table 1:** Demographic characteristics of study units (N=400)

Demographic characteristics	State	N (%)
Gender	Male	240(60)
	Female	160(40)
Age (years)	60-69	305(76.3)
	70-79	59(14.7)
	80 and beyond	36(9)
State of living	Alone	34(8.5)
	With partner	142(35.5)
	With partner and children	149(37.2)
	Other	75(18.8)
Education	Illiterate	349(87.2)
	High School	41(10.2)
	Diploma	10(2.5)
Marital Status	Married	299(74.8)
	Divorced	11(2.8)
	Widowed	90(22.5)
Financial Status	Poor	72(18)
	Average	250(62.5)
	Good	78(19.5)
		

**Table 2:** Mean score of quality of life of the elderly population covered by healthcare centers and bases in Tehran for each of the 8 determinants of quality of life

Indices of Quality of Life	Mean	SD
Physical Functioning (PF)	53.2	29.1
Role Physical(RP)	31.6	38.1
Bodily Pain(BP)	51	23.5
General Health(GH)	47.1	19.6
Vitality(V)	52.4	20.3
Social Functioning (SF)	60	30.3
Role Emotional(RE)	40.9	42.4
Mental Health(MH)	61.4	19.7
Physical Component(PC)	45.7	21.8
Mental Component(MC)	53.7	23.7
Overall Quality of Life(OQL)	49.7	20.6

We used the Kruskal-Wallis test to evaluate the impact of variables of age, gender, education, and financial status, status of living and marital status on the mean scores of indices of quality of life. The findings indicate that the variable of gender affects the mean scores of all indices of quality of life, with mean scores significantly lower for women compared to men except in the case of role physical. Moreover, the mean scores of all indices decreased significantly with increasing age, except in the case of vitality and mental health (Table 3).

**Table 3:** Relationship of variables of gender and age with mean scores of quality of life in population under study (N=400)

Indices of Quality of Life	Gender					Age						

	Female (N=160)		Male (N=240)		P Value	60-69		70-79		80 and beyond		P Value
	
	Mean	SD	Mean	SD		Mean	SD	Mean	SD	Mean	SD	
Physical Functioning	44.1	28.7	59.4	27.8	P<0.001	50	27.3	35.8	26.1	30	19	P<0.001
Role Physical	31.1	38.9	31.3	37.6	P<0.621	35	38	34.2	42.1	11.6	18.8	P<0.001
Bodily Pain	38.7	18.2	59.3	23.1	P<0.001	57.7	22.5	56.4	23.4	27.9	19.9	P<0.001
General Health	41.4	18.6	51	19.3	P<0.001	50	19.2	49.7	18.6	27.1	12.8	P<0.001
Vitality	46.1	17.7	56.6	20.8	P<0.001	53.4	21	48.7	18.2	45.1	16.9	P<0.283
Social Functioning	49.9	29.5	66.7	28.9	P<0.001	63.3	30.5	50.1	30.2	43.4	23.6	P<0.001
Role Emotional	31.7	41.1	47.1	42.3	P<0.001	45.1	41.8	35.6	38.9	35.2	45.7	P<0.001
Mental Health	54.7	19.3	65.9	18.7	P<0.001	67.1	21	64.8	15.6	62.6	9.8	P<0.002
Physical Component	39	21.7	50.2	20.7	P<0.001	46.9	20.7	44	21.3	21.7	15.3	P<0.001
Mental Component	45.6	21.7	59.1	23.5	P<0.001	53	25.3	49.8	18.7	49.1	14.9	P<0105
Overall Qualityof Life	42.3	19.4	54.6	20	P<0.001	52	21.1	46.9	18.7	35.4	11.5	P<0.001

An assessment of the relationship between the mean scores of indices of quality of life and education revealed significant relationships in all indices; for all indices, people with higher education scored higher compared to those who were illiterate. In addition, the variable of financial status affected all indices of quality of life with those people with good financial status scoring higher compared to the other two groups, and this difference was significant for all indices (Table 4). The variable of status of living influenced all indices of quality of life, with those people living with their partners and children scoring significantly higher in all indices compared to other groups (Table 5). Table 5 presents the relationship between mean scores of indices of quality of life and marital status, as determined by the Kruskal-Wallis test. For all indices, the married individuals scored significantly higher to those unmarried–divorced or widowed.

**Table 4:** Relationship of variables of education and financial status with mean scores of indices of quality of life in population under study (N=400)

Indices of Q o L	Education				Financial Status			

	Illiterate	High School	Diploma	P Value	Poor	Average	Good	P Value
	
	Mean (SD)	Mean (SD)	Mean (SD)		Mean (SD)	Mean (SD)	Mean (SD)	
PF	50.2(29.5)	72.8(13.8)	78.5(17.3)	p<0.001	30.6(32.6)	53.9(28)	63.9(24.6)	p<0.001
RP	27.6(38.4)	55.5(21.3)	71(14.1)	p<0.001	19.6(11.6)	28.3(36.8)	64.2(36.8)	p<0.001
B P	48.3(23)	67.3(19.2)	78.5(12.9)	p<0.001	30.3(20.2)	52(22.2)	64.3(20.5)	p<0.001
G H	43.9(17)	67.1(22.3)	80(15.1)	p<0.001	41.9(16.1)	47.5(20.6)	50.9(18.5)	p<0.32
V	49.9(19.8)	69.3(16.1)	69.5(12.1)	p<0.001	43.3(17.2)	53.8(21.1)	56.4(18.1)	p<0.001
S F	58(31)	71(19.3)	82.4(22.2)	p<0.005	45.1(35.8)	57.7(27.5)	80.9(21.4)	p<0.001
R E	38.9(42.8)	50.4(37.4)	73.3(30.6)	p<0.005	19.9(35.2)	38.9(42.3)	66.7(36)	p<0.001
M H	60.1(20)	69.5(14.6)	75.6(14.7)	p<0.001	55.9(22.1)	62.2(20.1)	64(14.6)	p<0.009
P C	42.5(20.9)	65.7(12.9)	77(10.4)	p<0.001	30.6(14)	45.4(21)	60.8(19.9)	p<0.001
M C	51.7(23.7)	65.7(21)	75.2(9.9)	p<0.001	41.1(22.4)	53.2(23.1)	67(20)	p<0.001
OQ L	47.1(20.4)	65.4(11.8)	76.1(8)	p<0.001	35.8(15.1)	49.3(19.9)	63.9(18.3)	p<0.001

**Table 5:** Relationship of variables of status of living and marital status with mean scores of indices of quality of life in population under study (N=400)

Indices of Qo L	Status of Living					Marital Status			

	Alone	With Partner	With Partner and Children	Other	P Value	Married	Divorced	Widowed	P Value
	
	Mean (SD)	Mean (SD)	Mean (SD)	Mean (SD)		Mean (SD)	Mean (SD)	Mean (SD)	
P F	40.3(32.5)	47.2(29.9)	62.1(24)	38.5(24.5)	P<0.001	56(27.4)	38.2(20)	32.8(25.3)	P<0.001
R P	27.8(35.6)	28.1(30.8)	50.5(39.3)	17.3(34.1)	P<0.001	37.1(38.6)	25.7(29)	17.9(31.6)	P<0.001
B P	52.7(22.2)	53.2(23.2)	58(24.4)	42(18.8)	P<0.001	59.5(22.7)	55.5(15.2)	34.4(19)	P<0.001
GH	50.1(26)	52(14)	56.7(20)	33(10)	P<0.001	52.1(19.1)	41.1(29.7)	32.7(8.6)	P<0.001
V	45.6(17.6)	47.1(17.6)	66.9(16)	36.6(14.1)	P<0.001	56.7(19.6)	40.9(23.6)	39.4(15.8)	P<0.001
SF	43.8(32)	45(30.3)	70.1(24.1)	50.2(31.9)	P<0.001	63.1(29.7)	55.7(16.2)	50(31.4)	P<0.003
R E	26.5(32.6)	27.5(40.6)	68.7(34.7)	19.6(34.3)	P<0.001	45.6(42.2)	36.4(31.5)	25.9(41.1)	P<0.001
MH	57.3(14.5)	59.4(23)	69.3(14.1)	49.7(17.9)	P<0.001	64(19.7)	62.6(26.7)	50.9(16.3)	P<0.001
P C	42.2(24.8)	43.9(18.1)	56.8(20.6)	32.7(18.6)	P<0.001	51.2(19.8)	40.1(19.8)	28.4(18.8)	P<0.001
MC	43.8(22)	46(23)	68.7(18.1)	39(19.5)	P<0.001	57.4(23.9)	48.9(17.1)	40.1(19.9)	P<0.001
OQ L	43(21.6)	44.9(17.2)	62.8(17.7)	35.9(17.3)	P<0.001	54.3(20)	44.5(16.9)	35.3(16.2)	P<0.001

## 4. Discussion

The present study uses the standardized tool SF-36 to evaluate the impact of demographic variables of gender, age, education, financial status, status of living and marital status on quality of life of the elderly population covered by healthcare centers and bases of Tehran Province.

Regarding the variable gender (Table 3), women scored significantly lower in all indices (except role physical) compared to men. This finding is consistent with previous studies (Farzianpour et al., 2012a, 2012b, 2015a, 2015b, 2016; Abdollahi et al., 2013; Rakhshan et al., 2014).

Nevertheless, one study failed to indicate gender to be an influential variable on quality of life of elderly population and indicated other factors, such as functional disabilities, to be more important (Nesbi et al., 2000; Farzianpour et al., 2014). Based on all these, it appears that the higher quality of life for men may be due to the fact that men are better provided with the facilities and benefits of the society. Furthermore, elderly women tend to be dependent on others financially and often lack an independent source of income, thus jeopardizing their status compared to men (Farzianpour et al., 2014; 2015b; Aghamolaei et al., 2010).

Regarding the impact of age variable on mean score of quality of life (Table 3), the mean scores of elderly individuals with lower age were significantly better for all indices compared to those with more advanced age. Thus, increasing age aggravates the quality of life in all dimensions. Evidently, advanced age is associated with greater occurrence of disabilities, manifesting as limitations in physical activities. The findings of the present study are consistent with those of previous study, with the exception of mental health in which older individuals scored negligibly higher compared to younger participants (Tsai et al., 2004; Sadat Vahdaninia et al., 2005; Physical Activity Guidelines Advisory Committee Report, 2010). Regarding the education variable, as Table 4 demonstrates, quality of life improves with education increasing from illiteracy to high school diploma. This tendency is observed for all dimensions of quality of life with a statistically significant relationship for all items. In general, it may be stated that improved education increases the awareness of individuals, thus enabling the educated elderly to have better control over the factors influencing their physical and mental health, resulting in an improved quality of life. On the other hand, higher education may be an indicator of better welfare, thus enhancing the quality of life in a multi-factorial fashion. In the dimension of social function, we observed that educated individuals tended to function better socially compared to their less educated peers. This may be accounted for by the fact that the educated elderly enjoy better opportunities for establishing friendships and acquaintances, resulting in an improved social function. Similarly, previous studies have indicated that higher education serves as a positive factor for a healthy elderly life (Hellstrom et al., 2004; Montazeri et al., 2005). Nonetheless, one study found no significant relationship between education and quality of life (Montazeri et al., 2005). As Table 4 illustrates, improvement of financial status from poor to good enhanced the quality of life significantly in all dimensions. Since financial status is an essential factor in most aspects of an individual’s life, it is capable of influencing the quality of life per se, as well as modifying other influential factors. This finding is consistent with those of a study by Tajvar et al. (2008). Regarding status of living (Table 5), our findings indicate that individuals living alone tended to have significantly lower scores on all dimensions of quality of life compared to individuals who lived with others – partner, children, etc. This correlation was expected, as previous studies had found similar results Tajvar et al. (Tajvar et al., 2008). As Table 5 demonstrates, married individuals scored significantly higher in all dimensions of quality of life compared to unmarried participants; a findings which is consistent with previous studies (Knurowski et al., 2004; Sadat Vahdaninia et al., 2005; Tajvar et al., 2008). Since isolation and loneliness constitute a potential major risk for health in senescence, it is crucial to provide the elderly population with a supportive atmosphere so that they may confront this factor appropriately (Meek et al., 2001). A feeling of belonging has been previously demonstrated to improve the quality of life in elderly people (Meek et al., 2001; Dechamps et al., 2010). Nevertheless, another study indicated that the overall quality of life of unmarried elderly women was higher compared to other marital statuses (Montazeri et al., 2005). However, the interference of the age factor must not be ignored; as the age advances, the number of people in the unmarried group rises. Furthermore, as mentioned above, advancement of age serves as a negative factor for quality of life. In conclusion, the findings of the present study indicate the predictive nature of health status and demographic variables for health-related quality of life. Considering the fact that during senescence, the quality of life may be easily jeopardized, it would be of utmost importance to envisage the background factors that influence the elderly quality of life.
